# Unsupervised Cardiac Image Segmentation via Multiswarm Active Contours with a Shape Prior

**DOI:** 10.1155/2013/909625

**Published:** 2013-10-02

**Authors:** I. Cruz-Aceves, J. G. Avina-Cervantes, J. M. Lopez-Hernandez, M. G. Garcia-Hernandez, M. A. Ibarra-Manzano

**Affiliations:** Universidad de Guanajuato, División de Ingenierías, Campus Irapuato-Salamanca, Carretera Salamanca-Valle de Santiago Km, 3.5+1.8 Km Comunidad de Palo Blanco, 36885 Salamanca, GTO, Mexico

## Abstract

This paper presents a new unsupervised image segmentation method based on particle swarm optimization and scaled active contours with shape prior. The proposed method uses particle swarm optimization over a polar coordinate system to perform the segmentation task, increasing the searching capability on medical images with respect to different interactive segmentation techniques. This method is used to segment the human heart and ventricular areas from datasets of computed tomography and magnetic resonance images, where the shape prior is acquired by cardiologists, and it is utilized as the initial active contour. Moreover, to assess the performance of the cardiac medical image segmentations obtained by the proposed method and by the interactive techniques regarding the regions delineated by experts, a set of validation metrics has been adopted. The experimental results are promising and suggest that the proposed method is capable of segmenting human heart and ventricular areas accurately, which can significantly help cardiologists in clinical decision support.

## 1. Introduction

 In clinical practice, magnetic resonance imaging (MRI) and computed tomography (CT) scanning are effective and widely used methods for the diagnosis and monitoring of cardiac disease. The process performed by a cardiologist on medical images consists of a visual examination followed by a manual delineation of the human organ, which can be subjective, susceptible to errors, and time consuming. Accordingly, the application of computational methods in order to obtain an efficient and accurate image segmentation for clinical decision support plays an essential role.

Automatic segmentation of human organs is an important and challenging task in medical image analysis. In recent years, several approaches have been reported for this purpose such as rule optimization with region growing in pelvic injuries [1], suppressed fuzzy c-means in brain magnetic resonance images [2], adaptive local multiatlas in human heart [3], graph cut in multiple human organs [4, 5] active contour models (ACM) in lungs from magnetic resonance images of the torso [6], intravascular ultrasound images [7], and human prostate [8]. 

ACM is an energy-minimizing spline curve consisting of a set of discrete control points known as snaxels. This spline is attracted towards features such as edges or lines through the evaluation of internal and external forces. ACM is prone to stagnate in local minima and is also highly sensitive to initialization because it requires to be close to the target object, otherwise failure of convergence will occur.

Since ACM was proposed by [9], many variations have been developed to improve these shortcomings through the introduction of some prior knowledge such as active shape models [10], ACM based on level set method [11], shape prior in left ventricle [12] and cerebellum [13], registered active shape model (RASM) in traumatic pelvic CT images [14], and ACM with particle swarm optimization (PSO) for womb fibroma in supersonic images [15], where static searching windows (size of 10 × 10 or 15 × 15 pixels) were dynamically generated depending on the initial position of the interactive control points. The performance of PSO approach is robust in local minima problem, and according to the tests, it provides an accurate medical image segmentation within an appropriate executing time.

PSO is a stochastic and population-based method inspired by the cognitive and social behavior of bird flocking to solve optimization problems in continuous spaces [16, 17]. This computational intelligence technique consists of a set of potential solutions known as swarm, where each potential solution is referred to as particle. In the PSO strategy, all the particles are guided by the best particle of the swarm, and each particle keeps track of its best solution found through iterations. Since being not computationally expensive and highly efficient to solve optimization problems, PSO has been used in a wide range of medical applications such as hepatitis disease diagnosis [18], tumor classification [19], and 3D medical registration [20].

Although the aforementioned algorithms provide satisfactory results regarding accuracy segmentation and noise sensitivity, more efforts are necessary to develop clinical decision support methods. In this paper, we introduce a new unsupervised image segmentation method based on particle swarm optimization and scaled active contours with shape prior. The proposed method uses PSO over a polar coordinate system to perform the segmentation task by increasing the exploration and exploitation capabilities with respect to the traditional ACM. In addition, this method utilizes the alignment process proposed in [21] to construct a target object template, which is used to determine the initial positioning of the polar coordinate system. Then, the template is scaled to a different size in order to generate potential solutions and assuming that the target object is confined within them. This proposed method addresses the segmentation problem of human heart and ventricular areas from CT and MR images, respectively. Furthermore, to evaluate the segmentation results with respect to regions outlined by experts and by different computational methods, a set of validation metrics is presented.

The remainder of this paper is organized as follows. In Section 2, we introduce the fundamentals of particle swarm optimization and active contour model with shape prior, along with the proposed segmentation method. The experimental results are discussed in Section 3, and conclusions are given in Section 4.

## 2. Materials and Methods

### 2.1. Overview of Particle Swarm Optimization (PSO)

 PSO is an artificial intelligence algorithm proposed by [16] and modified by [17] to solve optimization problems. PSO consists of a swarm of potential solutions called particles. Each particle is viewed as a point in an *N*-dimensional space *X*_*i*_ = {*x*_*i*1_, *x*_*i*2_,…, *x*_*iN*_}. During each iteration, every particle moves through hyperspace to a new position according to the following velocity equation:

(1) vi(t+1)=φvi(t)+κr1(pbest−xi(t))+κr2(pgbest−xi(t)),

where *x*_*i*_ is the current particle in the time step (*t*) and *v*_*i*_(*t*) represents its velocity, *φ* is the inertia weight, *r*_1_, *r*_2_ ~ *U*(0,1) where *U* is a uniform distribution, *κ* represents the learning factor, *p*_*best*_ is the best solution found by the current particle, and *p*_*gbest*_ is the best solution found by the best particle of the whole swarm. On the other hand, assuming that the new velocity of the current particle has been updated, (2) is used to compute its new position within the search space:

(2) $$\begin{array}{c} x_{i}\left(t+1\right)=x_{i}\left(t\right)+v_{i}\left(t+1\right). \end{array}$$

According to the above description, the PSO algorithm can be implemented through the following procedure.

1. Establish the swarm size and randomly initialize the position and velocity of each particle.
2. Evaluate each particle in the fitness function in order to update its *p*_*best*_, if the new fitness is better.
3. Find the best particle in the whole swarm and update *p*_*gbest*_, if the fitness value found is better.
4. Stop if the convergence criterion is satisfied (e.g., stability or number of iterations).
5. Update velocity and position of all the particles using (1) and (2), respectively, then repeat steps (2)–(5).

### 2.2. Active Contour Model with Shape Prior

 The conventional active contour model is a parametric curve that is driven by internal and external forces to minimize its energy function [9]. In order to incorporate a shape prior constraint within the active contour, the Chan and Vese method [11] is used, and it is defined by using the following equation:

(3) $$\begin{array}{c} E_{T}=w_{1}E_{1}+w_{2}E_{2}+w_{3}E_{3}, \end{array}$$

where *E*_*T*_ is the total energy composed of the energies *E*_1_, *E*_2_ and *E*_3_ and the weighting factors of the shape energy *w*_1_, *w*_2_ and *w*_3_. The first energy *E*_1_ is the active contour, which can be represented as

(4) E1=∫Ω((I−c1)2H(ϕ))dx dy+∫Ω((I−c2)2(1−H(ϕ)))dx dy+μ∫Ω|∇H(ϕ)|dx dy+ν∫ΩH(ϕ)dx dy,

where *Ω* is the image domain, *H*(·) is the Heaviside function, *I* is the image intensity, ∇ is the gradient operator, *μ* and *ν* are the weighting parameters of the length and area energies of the contour, *ϕ* is a signed distance function, and *c*_1_ and *c*_2_ are the mean intensity of the object and background, and they are given by the following equations:

(5) $$\begin{array}{c} c_{1}=\frac{\int_{\Omega }I\left(x,y\right)H\left(\phi \right)dx\;dy}{\int_{\Omega }H\left(\phi \right)dx\;dy}, \end{array}$$

(6) $$\begin{array}{c} c_{2}=\frac{\int_{\Omega }I\left(x,y\right)\left(1-H\left(\phi \right)\right)dx\;dy}{\int_{\Omega }\left(1-H\left(\phi \right)\right)dx\;dy}. \end{array}$$

*E*_2_ is the shape energy defined by the difference between the active curve and the shape template. This energy will be minimized by optimizing the transformation parameters, and it is expressed as follows:

(7) $$\begin{array}{c} E_{2}=\int_{\Omega }{\left(H\left(\phi \right)-H\left(\varphi_{T}\left(B^{T}\right)\right)\right)}^{2}dx\;dy, \end{array}$$

where *φ*_*T*_ represents the deformed template and *B* ^*T*^ is defined in a transformation matrix consisting of translation [*t*_*x*_, *t*_*y*_]^*T*^, scaling [*s*], and rotation [*θ*] parameters as follows:

(8) $$\begin{array}{c} B^{T}=\underset{M(a,b)}{\underset{︸}{\left[\begin{array}{ccc} 1 & 0 & t_{x}\\ 0 & 1 & t_{y}\\ 0 & 0 & 1 \end{array}\right]}}\times \underset{H(s)}{\underset{︸}{\left[\begin{array}{ccc} s & 0 & 0\\ 0 & s & 0\\ 0 & 0 & 1 \end{array}\right]}}\times \underset{R(\theta )}{\underset{︸}{\left[\begin{array}{ccc} \cos\theta & -\sin\theta & 0\\ \sin\theta & \cos\theta & 0\\ 0 & 0 & 1 \end{array}\right]}}, \end{array}$$

where *s* is a scaling factor, *θ* is the rotation angle parameter, and [*t*_*x*_, *t*_*y*_]^*T*^ are translation parameters in the horizontal and vertical axes. Finally, the third energy term *E*_3_ is the image-based force, which is the difference energy, and it is computed as

(9) $$\begin{array}{c} E_{3}=\int_{\Omega }{\left(\nabla H\left(\phi \right)-\nabla I\right)}^{2}dx\;dy. \end{array}$$

The three energies of the active contour are iteratively evaluated, and the contour evolution terminates when the difference between the previous and current segmented area becomes stable.

### 2.3. Proposed Image Segmentation Method

**Figure 1:**
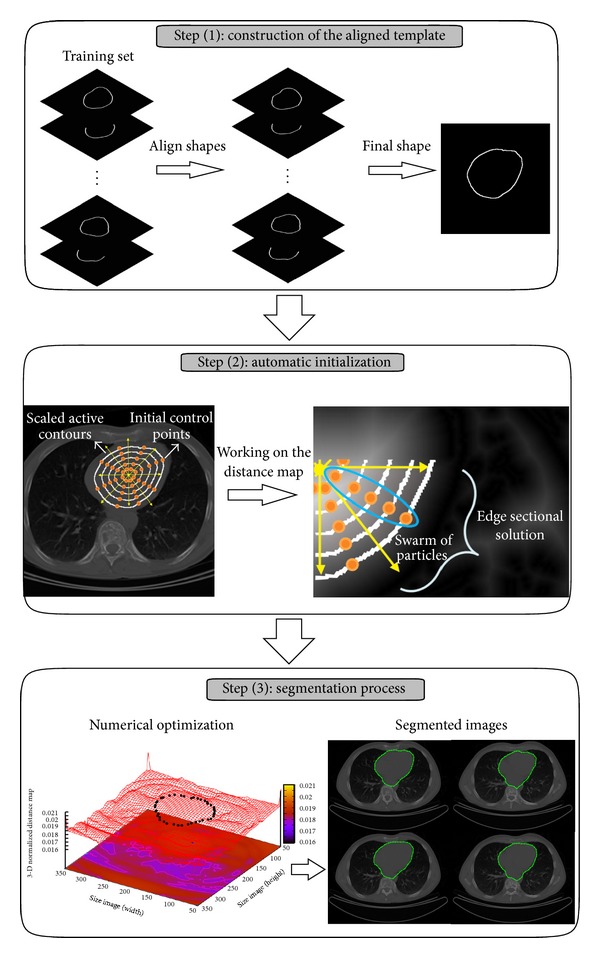
Process of the proposed image segmentation method.

 The proposed image segmentation method consists of three main steps as shown in Figure 1, and they are described below.

#### 2.3.1. Shape Representation and Construction of the Aligned Template

**Figure 2:**
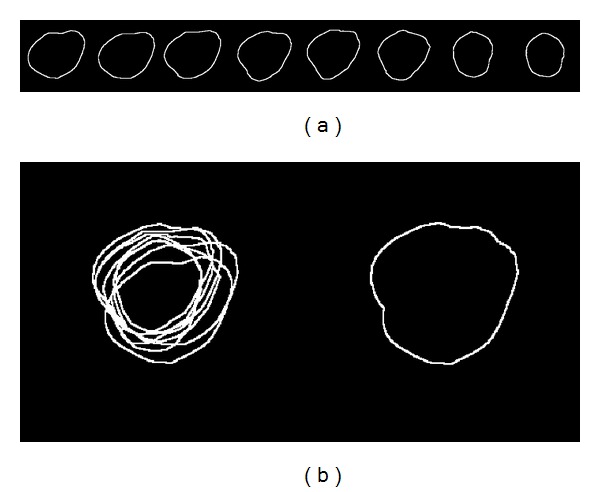
Training data: (a) eight 2D binary shape models of the human heart delineated by experts and (b) superposition of training binary shapes and contour obtained before alignment.

 In order to generate a template of the target object, a set of selected reference images is aligned, which leads to differences in position, direction, and scale. The aim of this step is to obtain a shape template through the alignment of a set of manually segmented images, which contain the human heart and ventricular areas. In Figure 2, to analyze the alignment procedure, a training set consisting of 8 human hearts is presented.

By using the technique developed in [21], we compute the shape alignment by estimating the parameters [*a*, *b*, *s*, *θ*]^*T*^ through (10), where *M*(*a*, *b*) is the *x* and *y* translation, *s* is the scale parameter, and *θ* is the rotation angle:

(10) $$\begin{array}{c} \left[\begin{array}{c} \tilde{x}\\ \tilde{y}\\ 1 \end{array}\right]=M\left(a,b\right)\times H\left(s\right)\times R\left(\theta \right)\times \left[\begin{array}{c} x\\ y\\ 1 \end{array}\right]. \end{array}$$

**Figure 3:**
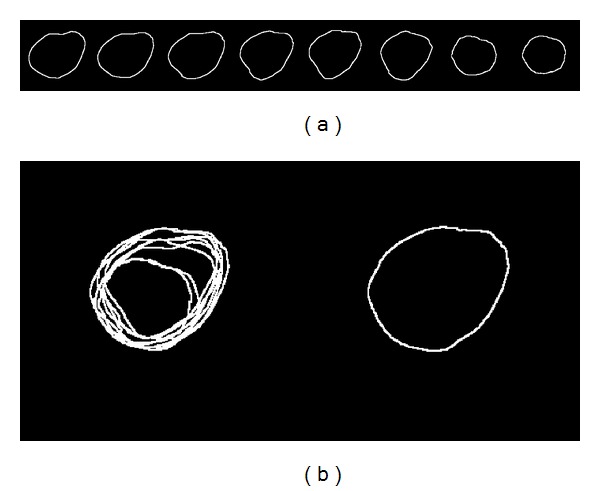
Shape templates: (a) aligned eight 2D shape models of the human heart and (b) superposition of the aligned binary shapes and contour obtained after alignment.

The product of the translation matrix *M*(*a*, *b*), scaling matrix *H*(*s*), and the in-plane rotation matrix *R*(*θ*) maps the coordinates (*x*, *y*) ∈ ℝ^2^ to coordinates $(\tilde{x},\tilde{y})\in {\mathbb{R}}^{2}$. Following the alignment process, the gradient descent method is used to minimize the following energy function:

(11) $$\begin{array}{c} E_{alig}=\overset{n}{\underset{i=1}{\sum }}\;\overset{n}{\underset{j=1,j\neq i}{\sum }}\left\{\frac{\int \int_{\Omega }^{}{\left({\tilde{I}}^{i}-{\tilde{I}}^{j}\right)}^{2}dA}{\int \int_{\Omega }^{}{\left({\tilde{I}}^{i}+{\tilde{I}}^{j}\right)}^{2}dA}\right\}, \end{array}$$

where $\tilde{I}$ is the transformed image based on the shape parameters, and *Ω* is the image domain. The gradient of *E*_*alig*_ in (11) is iteratively evaluated until convergence. The alignment results presented in Figure 3(a) are slightly different from the training binary shapes, and in Figure 3(b), the resulting maximum contour after alignment presents a significant variation regarding the maximum contour shown in Figure 2(b).

After adjusting each shape parameter, the final aligned template is obtained by superimposing all transformed images, and then, the maximum shape boundary is acquired.

#### 2.3.2. Multiswarm Initialization and Numerical Optimization

 The initialization stage is performed on the resulting distance map, where the origin of the polar coordinate system is automatically determined by using the maximum mutual information between the template and the current medical image. The coordinate system divides the target object through *θ* = 2*π*/*g*, where *g* denotes the number of degrees of each constrained polar section *S*. In our approach, parameters play an important role, which are described as follows number of scaled contours; this parameter has to be considered assuming that the target object is confined within the region of the initial contours. Number of control points determines the number of polar sections in which the target object is divided. Number of iterations is used to obtain proper segmentation results through the evaluation of the fitness function. Inertia weight controls the exploration and exploitation abilities of the swarm by weighing the previous velocity value on the new velocity and the learning factor parameter to scale the step size of the search. The numerical optimization is performed after the *n* scaled contours are produced and the *n* control points (particles) are generated for each constrained polar section *S*_*i*_, in which one edge sectional solution and one swarm of particles *O*_*i*_ must exist. PSO strategy is applied in each polar section *S*_*i*_ separately in order to be placed on its corresponding edge sectional solution. All the particles are evaluated according to the fitness function derived from the distance map, where through iterations the best particle *g* *best* of each swarm is updated only if a best particle is found in its constrained search space. When the optimization process for each swarm is finished, the resulting segmented object is acquired by connecting the *g* *best* particle of each swarm to each other.

The proposed method presents the following advantages: (1) the initial contours are automatically initialized according to the shape template obtained from the alignment process; (2) the number of discrete points can be adjusted directly by modifying the number of polar sections through the *g* parameter. Due to these advantages, the proposed method can be extended to work with sequential CT and MR images by just applying the maximum mutual information to reproduce the coordinate system on the set of images. 

The procedure of the proposed image segmentation method is described as follows.

1. Align predefined shapes according to [21] and obtain template after alignment.
2. Perform maximum mutual information to initialize coordinates (*x*, *y*) of the polar coordinate system.
3. Initialize degrees *g* and number of snakes.
4. Initialize the PSO parameters: number of iterations, inertia weight, and learning factor.
5. Generate one swarm of particles for each polar section *S*_*i*_ assigning the current control points as particles.
6. For each swarm *O*_*i*_, initialize velocities and assign the initial *p* *best* and *g* *best*.
  - Apply restriction of the search space to ignore improper particles.
  - Evaluate each particle in fitness function derived from the distance map.
  - Update *p* *best* and *g* *best* if better particles are found.
  - Apply (1) and (2), respectively.
  - Stop if the convergence criterion is satisfied (e.g., stability or number of iterations), otherwise go to step (a).

### 2.4. Evaluation Metrics

 In order to assess the performance of the proposed method, the maximum cardinality similarity metric, Hausdorff distance, Jaccard, Dice, and correlation indices have been adopted to compare the segmentation results between the regions outlined by experts and the regions obtained by computational methods:

(12) $$\begin{array}{c} J\left(A,B\right)=\frac{A\cap B}{A\cup B}, \end{array}$$

(13) $$\begin{array}{c} D\left(A,B\right)=\frac{2\left(A\cap B\right)}{A+B}, \end{array}$$

(14) $$\begin{array}{c} \rho \left(A,B\right)=\frac{\mathrm{cov}\left(A,B\right)}{\sigma_{A}\sigma_{B}}. \end{array}$$

The Jaccard *J* (*A*, *B*) and Dice *D* (*A*, *B*) indices are computed using (12) and (13), and they are used as measures of similarity between reference (*A*) and automatic (*B*) segmented objects [13]. If regions *A* and *B* are entirely overlapping, the obtained result is 1, and it is 0 when these two regions are completely different. In addition, the correlation index (14) measures the linear relationship between the reference and automatic segmentations, and it is defined in the range [−1,1]. A correlation of 1 means perfect positive linear relationship, and −1 means negative linear relationship.

On the other hand, the Hausdorff distance is a metric for shape matching in medical image segmentation, which measures the similarity between two superimposed sets via (15), where *a* and *b* are the edge pixels in sets *A* and *B*, respectively, and ||*a* − *b*|| is the Euclidean distance:

(15) $$\begin{array}{c} H\left(A,B\right)=\underset{a\in A}{\max}\;\underset{b\in B}{\min}||a-b||. \end{array}$$

The maximum cardinality similarity metric is used in template-matching applications with a low computational complexity [22]. It is applied to the edge pixels of segmented objects using (16), where TP is the number of true positives, FN is the false negatives, and *N* is the total number of pixels in the image:

(16) $$\begin{array}{c} R_{n}=\left(\frac{N}{N-\text{TP}}\right)\left(\frac{\text{TP}}{\text{TP}+\text{FN}}-\frac{\text{TP}}{N}\right) \end{array}$$

## 3. Experimental Results

 In this section, we evaluate the performance of the proposed image segmentation method for segmenting the human heart and ventricular areas on CT and MR images, respectively. The computational implementations were performed using the GNU Compiler Collection (C++) version 4.4.5 running on Debian GNU/Linux 6.0, Intel Core i3 with 2.13 Ghz and 4 Gb of memory.

**Figure 4:**
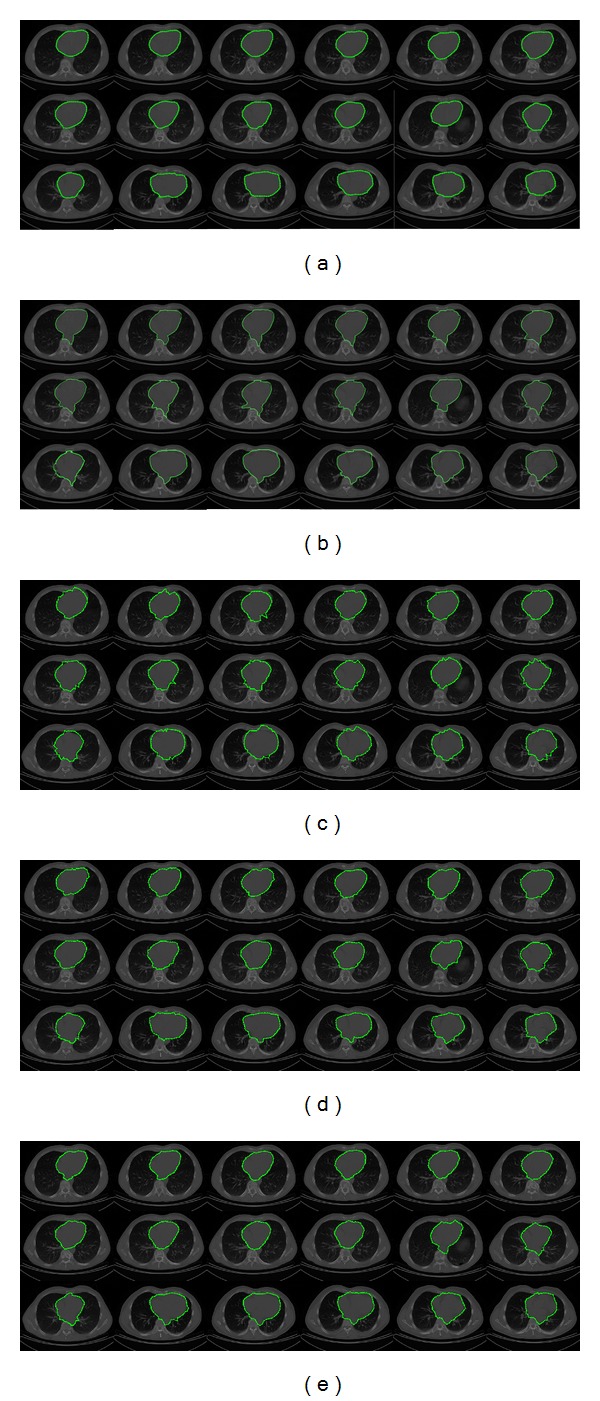
CT images (human heart segmentation): (a) manual delineation by experts, (b) results of Graph Cut method, (c) results of traditional ACM, (d) results of interactive Tseng method, and (e) results of proposed implementation.

In Figure 4, the segmentation results on a subset of CT images containing the human heart are presented. The whole dataset consists of 144 CT images of size 512 × 512 pixels obtained from different patients. In Figure 4(a), the human heart outlined by cardiologists is presented.  Figure 4(b) illustrates the segmentation results obtained through the interactive Graph Cut method [5], which were computed with an average executing time of 0.185 s per image. In Graph Cut method, the experts defined the human heart area and the background seeds in an interactive way. In Figure 4(c) the segmentation results by using the traditional ACM are presented, in which the noise sensitivity and fitting problem are shown. The ACM parameters were determined according to the multiple objects test presented by [15] as 45 control points, *α* = 0.01, *β* = 0.9, and *γ* = 0.05, obtaining in our test an average executing time of 0.157 s per image.  Figure 4(d) shows the human heart segmentations obtained with the interactive Tseng method. The parameters of this method were determined according to [15], and they were set as 45 control points, window size as 30 × 30 pixels, and 9 particles for each swarm, given an average executing time of 0.176 s per image. Finally, in Figure 4(e), the segmented images by using the proposed method reveal an appropriate human heart segmentation avoiding the local minima problem. In this simulation, the parameters were set as number of scaled contours = 9, number of snaxels = 45, iterations = 10, inertia weight = 0.5, and learning factor = 0.9, obtaining an average executing time of 0.198 s per image. In our approach, the learning factor and inertia weight were tuned experimentally taking into account the following two considerations: firstly, the number of different potential solutions generated through the iterations and secondly, by considering the number of improper solutions in order to perform local exploitation instead exploration [23, 24].

From the dataset of CT images described above, in Table 1, a subset of the segmentation results is presented. These results were obtained by comparing the regions delineated by experts with the segmentation results of the Graph Cut, ACM, Tseng method, and our proposed method. Based on the similarity analysis, the human heart segmentation results suggest that the proposed method can lead to more efficiency in the presence of concavities and noise than the comparative interactive methods.

**Figure 5:**
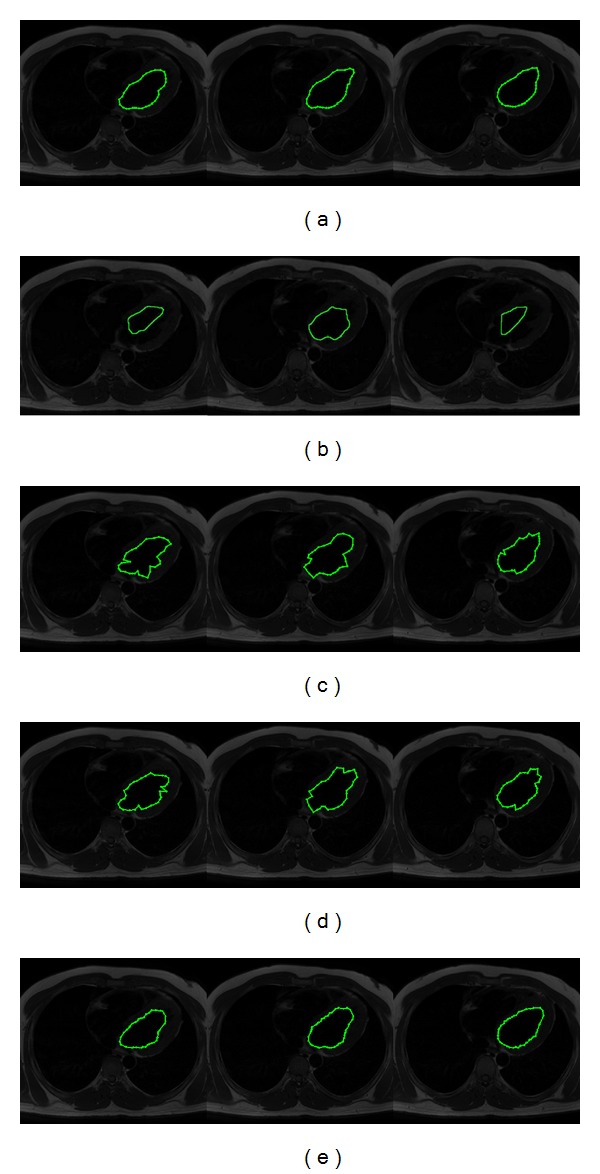
MR images (left ventricle segmentation): (a) manual delineation by experts, (b) results of Graph Cut method, (c) results of traditional ACM, (d) results of interactive Tseng method, and (e) results of proposed implementation.

Figure 5 presents the segmentation results on a subset of MR images containing the human left ventricle. These images have been extracted from a previously segmented dataset with 23 MR images of size 512 × 512 pixels. In Figure 5(a), the human left ventricle delineated by cardiologists in different test images is presented.  Figure 5(b) shows the segmentation results obtained in an average executing time of 0.166 s by using the interactive Graph Cut method, in which experts defined the human left ventricle area and the background image interactively. These segmentation results cannot be adjusted to the correct left ventricle boundary, which is improved by the next three methods described below.  Figure 5(c) illustrates the segmentation results through the traditional ACM, where the local minima problem is clearly shown. The ACM parameters were adjusted according to the medical tests presented in [15], and they were set as 35 control points, *α* = 0.013, *β* = 0.845, and *γ* = 0.195, obtaining an average executing time of 0.108 s per image. In Figure 5(d), the human heart segmentation results by applying the interactive Tseng method are illustrated. The parameters of this simulation were chosen according to supersonic image test presented by [15] as 35 control points, window size as 30 × 30 pixels, and 15 particles for each swarm, given an average executing time of 0.209 s per image. Moreover, Figure 5(e) presents the segmentation results acquired by the proposed method. The segmented images show in a suitable way the boundary of the human left ventricle avoiding the noise sensitivity problem. The parameters of this experiment were set as number of scaled contours = 7, number of snaxels = 35, iterations = 10, inertia weight = 0.4, and learning factor = 0.7, obtaining an average executing time of 0.169 s per image.

According to the left ventricle dataset of MR images previously described, in Table 2, the average of the segmentation results obtained by Graph Cut, ACM, Tseng method, and our proposed method is compared with the regions outlined by experts. This similarity analysis shows that the proposed methods perform the human left ventricle segmentation accurately with respect to the other computational methods.

**Figure 6:**
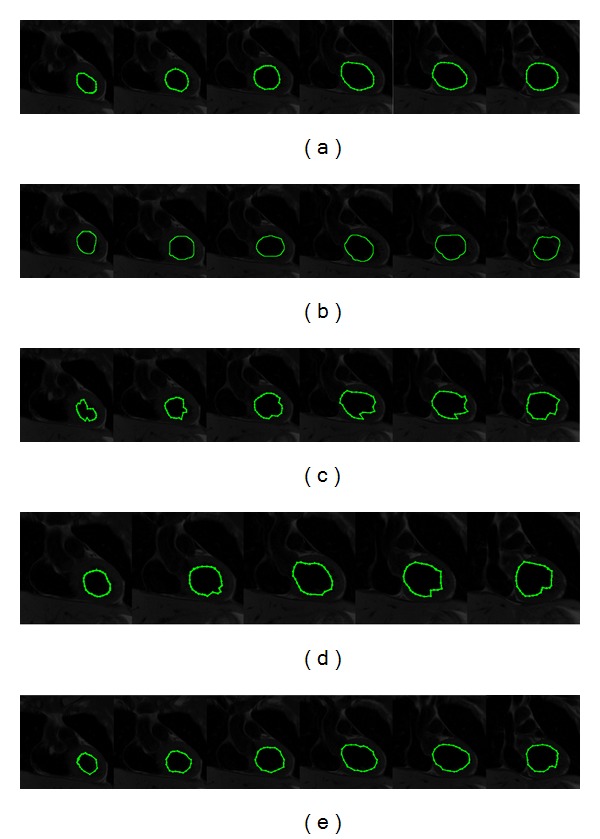
MR images (ventricular area segmentation): (a) manual delineation by experts, (b) results of Graph Cut method, (c) results of traditional ACM, (d) results of interactive Tseng method, and (e) results of proposed implementation.

 In Figure 6, the segmentation results on a subset of coronal MR images containing ventricular areas are presented. These images have been extracted from a dataset of 19 images with size 256 × 256 pixels. In Figure 6(a), the manual delineations made by experts on the ventricular area are illustrated.  Figure 6(b) presents the segmentation results obtained with the interactive Graph Cut, which were acquired with an average executing time of 0.192 s per image. This method fails to fit the ventricular area boundary because of the presence of noise. In Figure 6(c), the traditional ACM is applied, where the segmented ventricular areas cannot fit the true boundary accurately. The ACM parameters were set according to the medical image test introduced by [15] as 25 control points, *α* = 0.013, *β* = 0.845, and *γ* = 0.195, obtaining in our tests an average executing time of 0.095s per image. Consequently, in Figure 6(d), the ventricular area segmentation results through the interactive Tseng method are shown. The parameters of this simulation were tuned according to the medical image test presented by [15] as 25 control points, window size as 30 × 30 pixels, and 15 particles for each swarm, given an average executing time of 0.143 s per image.  Figure 6(e) shows the segmentation results obtained by the proposed method, where the segmented images fit the true ventricular area boundary more accurately than the previous computational methods. The parameters of this simulation were set as number of scaled contours = 7, number of snaxels = 25, iterations = 10, inertia weight = 0.4, and learning factor = 0.7, achieving an average executing time of 0.154 s per image.

In Table 3, the average of the previously segmented ventricular areas through the Graph Cut, ACM, Tseng method, and our proposed method is compared to the regions outlined by experts. The performed similarity analysis suggests that the proposed method is more robust in ventricular area segmentation than the other computational methods according to the Jaccard and Dice indices with 85% and 92%, respectively.

Segmentation results were compared with the regions outlined by cardiologists on CT and MR images in the aforementioned datasets. As shown in similarity analysis, the proposed method is able to detect human cardiac organs with a high accuracy and effectiveness. Moreover, it can help cardiologists to better analyze the medical images and increase their monitoring abilities.

## 4. Conclusions

 We have presented a new unsupervised image segmentation method based on particle swarm optimization and scaled active contours with shape prior. This method generates different scaled contours according to the shape template obtained from an alignment process. Then, particle swarm optimization is used to perform the segmentation task over constrained polar sections. This novel approach was applied to segment the human heart and ventricular areas from CT and MR images. In order to evaluate the segmentation results acquired through the proposed method, correlation and Jaccard and Dice indices were used. According to the experimental results, the proposed method proves to be capable of segmenting human heart and ventricular areas accurately compared to the segmentations acquired by Grap Cut, traditional ACM, and interactive Tseng method. In addition, the experimental results have also shown that the exploitation capability of the proposed method is highly appropriate for cardiac medical image applications.

## Figures and Tables

**Table 1:** Similarity measure with the Jaccard and Dice indices, Hausdorff distance, and the Maximum cardinality similarity metric among the regions segmented by the Graph Cut method, traditional ACM, interactive Tseng method, our proposed method, and the regions outlined by experts of the CT dataset.

Test	Graph Cut versus experts				ACM versus experts				Tseng versus experts				Our method versus experts			
Image	(J)	(D)	(H)	(MCSM)	(J)	(D)	(H)	(MCSM)	(J)	(D)	(H)	(MCSM)	(J)	(D)	(H)	(MCSM)
1	0.551	0.711	4.000	0.343	0.698	0.822	10.049	0.681	0.636	0.777	10.198	0.781	0.836	0.911	1.036	0.759
5	0.698	0.822	5.385	0.497	0.636	0.777	5.099	0.396	0.666	0.800	10.000	0.644	0.800	0.888	3.989	0.833
10	0.607	0.755	3.605	0.374	0.607	0.755	4.472	0.452	0.578	0.733	5.000	0.684	0.764	0.866	1.000	0.813
15	0.475	0.644	10.000	0.512	0.836	0.911	6.580	0.695	0.800	0.888	7.211	0.422	0.875	0.933	2.719	0.793
20	0.428	0.600	2.828	0.449	0.875	0.933	7.214	0.660	0.836	0.911	10.000	0.552	0.914	0.955	3.105	0.764
25	0.800	0.888	4.900	0.475	0.914	0.955	5.000	0.723	0.698	0.822	7.000	0.511	0.764	0.866	1.381	0.729
30	0.730	0.844	2.236	0.386	0.607	0.755	2.828	0.621	0.636	0.777	5.385	0.748	0.730	0.844	2.828	0.790
35	0.636	0.777	5.000	0.550	0.551	0.711	1.000	0.720	0.875	0.933	2.828	0.781	0.914	0.955	1.082	0.920
40	0.607	0.755	4.500	0.493	0.500	0.666	4.123	0.740	0.800	0.888	2.000	0.755	0.875	0.933	6.000	0.797
45	0.525	0.688	10.414	0.500	0.525	0.688	5.000	0.617	0.607	0.755	3.000	0.835	0.636	0.777	1.082	0.863
50	0.451	0.622	9.798	0.469	0.698	0.822	18.384	0.601	0.730	0.844	1.414	0.519	0.698	0.822	2.828	0.749
55	0.428	0.600	6.082	0.434	0.764	0.866	12.529	0.419	0.800	0.888	1.000	0.430	0.836	0.911	5.099	0.645
60	0.764	0.866	9.848	0.439	0.666	0.800	2.236	0.692	0.730	0.844	1.000	0.509	0.764	0.866	1.000	0.778
65	0.875	0.933	11.313	0.394	0.914	0.955	8.000	0.616	0.875	0.933	8.000	0.615	0.800	0.888	8.000	0.635
70	0.451	0.622	16.278	0.467	0.525	0.688	1.000	0.688	0.607	0.755	3.000	0.505	0.578	0.733	5.236	0.863
75	0.500	0.666	19.798	0.484	0.551	0.711	2.236	0.561	0.525	0.688	2.828	0.683	0.607	0.755	2.828	0.655
80	0.551	0.711	14.866	0.398	0.607	0.755	5.000	0.504	0.578	0.733	2.000	0.879	0.666	0.800	5.000	0.843
85	0.578	0.733	12.236	0.394	0.666	0.800	3.162	0.542	0.698	0.822	3.083	0.827	0.730	0.844	4.885	0.737
90	0.698	0.822	6.403	0.468	0.764	0.866	4.123	0.511	0.800	0.888	9.433	0.534	0.764	0.866	6.336	0.832
95	0.764	0.866	13.605	0.502	0.836	0.911	11.401	0.567	0.836	0.911	5.099	0.687	0.800	0.888	8.000	0.904
100	0.525	0.688	14.123	0.467	0.578	0.733	1.000	0.576	0.551	0.711	1.000	0.579	0.636	0.777	2.236	0.878
105	0.406	0.577	1.414	0.523	0.475	0.644	13.601	0.604	0.607	0.755	5.385	0.632	0.607	0.755	1.414	0.838
110	0.384	0.555	6.0	0.602	0.428	0.600	5.000	0.691	0.500	0.666	7.000	0.575	0.551	0.711	8.000	0.776
115	0.836	0.911	8.944	0.586	0.800	0.888	13.038	0.461	0.836	0.911	4.472	0.523	0.875	0.933	4.242	0.869
120	0.764	0.866	9.848	0.514	0.836	0.911	15.231	0.695	0.764	0.866	6.000	0.663	0.836	0.911	6.000	0.817
125	0.666	0.800	13.601	0.611	0.875	0.933	14.142	0.609	0.914	0.955	6.708	0.718	0.956	0.977	3.000	0.948
130	0.578	0.733	10.770	0.598	0.607	0.755	12.649	0.677	0.875	0.933	7.280	0.618	0.914	0.955	4.123	0.843
135	0.698	0.822	11.401	0.487	0.698	0.822	17.720	0.741	0.956	0.977	5.000	0.681	0.956	0.977	2.236	0.705
140	0.636	0.777	8.540	0.534	0.764	0.866	11.704	0.588	0.836	0.911	1.000	0.653	0.875	0.933	2.000	0.834
144	0.525	0.688	6.827	0.568	0.578	0.733	7.280	0.619	0.730	0.844	10.000	0.843	0.914	0.955	1.000	0.935

Average	0.607	0.755	8.485	0.546	0.666	0.800	7.182	0.692	0.764	0.866	5.716	0.778	0.875	0.933	5.228	0.856

**Table 2:** (Left ventricle) Average similarity measure with the correlation, Jaccard and Dice indices, Hausdorff distance, and the maximum cardinality similarity metric among the regions segmented by the Graph Cut method, traditional ACM, interactive Tseng method, our proposed method, and the regions outlined by experts of the MR dataset.

Comparative	Similarity measure				
Studies	(C)	(J)	(D)	(H)	(MCSM)
Graph Cut versus experts	0.7169	0.4893	0.6571	12.623	0.556
ACM versus experts	0.8608	0.5909	0.7428	9.501	0.637
Tseng versus experts	0.8688	0.7500	0.8571	7.284	0.0.695
Our method versus experts	0.8866	0.8421	0.9142	6.476	0.711

**Table 3:** Average similarity measure with the correlation, Jaccard and Dice indices, Hausdorff distance, and the maximum cardinality similarity metric among the ventricular areas segmented by the Graph Cut method, traditional ACM, interactive Tseng method, our proposed method, and the regions outlined by experts of the MR dataset.

Comparative	Similarity measure				
Studies	(C)	(J)	(D)	(H)	(MCSM)
Graph Cut versus experts	0.8027	0.5151	0.68	6.458	0.446
ACM versus experts	0.8153	0.6129	0.76	5.962	0.514
Tseng versus experts	0.8384	0.7857	0.88	3.841	0.623
Our method versus experts	0.8709	0.8518	0.92	2.781	0.706

## References

[1] Davuluri P, Wu J, Tang Y (2012). Hemorrhage detection and segmentation in traumatic pelvic injuries. *Computational and Mathematical Methods in Medicine*.

[2] Nyma A, Kang M, Kwon Y, Kim C, Kim J (2012). A hybrid technique for medical image segmentation. *Journal of Biomedicine and Biotechnology*.

[3] van Rikxoort EM, Isgum I, Arzhaeva Y (2010). Adaptive local multi-atlas segmentation: application to the heart and the caudate nucleus. *Medical Image Analysis*.

[4] Boykov Y, Jolly M Interactive organ segmentation using graph cuts.

[5] Schmidt FR, Töppe E, Cremers D Efficient planar graph cuts with applications in computer vision.

[6] Middleton I, Damper RI (2004). Segmentation of magnetic resonance images using a combination of neural networks and active contour models. *Medical Engineering and Physics*.

[7] Zhu X, Zhang P, Shao J, Cheng Y, Zhang Y, Bai J (2011). A snake-based method for segmentation of intravascular ultrasound images and its in vivo validation. *Ultrasonics*.

[8] Liu X, Haider MA, Yetik IS (2011). Unsupervised 3D prostate segmentation based on diffusion-weighted imaging MRI using active contour models with a shape prior. *Journal of Electrical and Computer Engineering*.

[9] Kass M, Witkin A, Terzopoulos D (1988). Snakes: active contour models. *International Journal of Computer Vision*.

[10] Cootes TF, Taylor CJ, Cooper DH, Graham J (1995). Active shape models-their training and application. *Computer Vision and Image Understanding*.

[11] Chan TF, Vese LA (2001). Active contours without edges. *IEEE Transactions on Image Processing*.

[12] Liu W, Shang Y, Yang X, Deklerck R, Cornelis J (2011). A shape prior constraint for implicit active contours. *Pattern Recognition Letters*.

[13] Hwang J, Kim J, Han Y, Park H (2011). An automatic cerebellum extraction method in T1-weighted brain MR images using an active contour model with a shape prior. *Magnetic Resonance Imaging*.

[14] Wu J, Davuluri P, Ward KR, Cockrell C, Hobson R, Najarian K (2012). Fracture detection in traumatic pelvic CT images. *International Journal of Biomedical Imaging*.

[15] Tseng C-C, Hsieh J-G, Jeng J-H (2009). Active contour model via multi-population particle swarm optimization. *Expert Systems with Applications*.

[16] Eberhart R, Kennedy J New optimizer using particle swarm theory.

[17] Shi Y, Eberhart R Modified particle swarm optimizer.

[18] Neshat M, Sargolzaei M, Toosi A, Masoumi A (2012). Hepatitis disease diagnosis using hybrid case based reasoning and particle swarm optimization. *ISRN Artificial Intelligence*.

[19] Abdi MJ, Hosseini S, Rezghi M (2012). A novel weighted support vector machine based on particle swarm optimization for gene selection and tumor classification. *Computational and Mathematical Methods in Medicine*.

[20] Lin C, Mimori A, Chen Y (2012). Hybrid particle swarm optimization and its application to multimodal 3d medical image registration. *Computational Intelligence and Neuroscience*.

[21] Tsai A, Yezzi A, Wells W (2003). A shape-based approach to the segmentation of medical imagery using level sets. *IEEE Transactions on Medical Imaging*.

[22] Correa-Tome F, Sanchez-Yanez R, Ayala-Ramirez V (2012). Measuring empirical discrepancy in image segmentation results. *IET Computer Vision*.

[23] Jiang M, Luo YP, Yang SY (2007). Stochastic convergence analysis and parameter selection of the standard particle swarm optimization algorithm. *Information Processing Letters*.

[24] van den Bergh F, Engelbrecht AP (2006). A study of particle swarm optimization particle trajectories. *Information Sciences*.

